# Genomic analysis of an emerging multiresistant *Staphylococcus aureus *strain rapidly spreading in cystic fibrosis patients revealed the presence of an antibiotic inducible bacteriophage

**DOI:** 10.1186/1745-6150-4-1

**Published:** 2009-01-13

**Authors:** Jean-Marc Rolain, Patrice François, David Hernandez, Fadi Bittar, Hervé Richet, Ghislain Fournous, Yves Mattenberger, Emmanuelle Bosdure, Nathalie Stremler, Jean-Christophe Dubus, Jacques Sarles, Martine Reynaud-Gaubert, Stephanie Boniface, Jacques Schrenzel, Didier Raoult

**Affiliations:** 1 URMITE CNRS-IRD UMR 6236, Faculté de Médecine et de Pharmacie, Université de la Méditerranée, 27 Boulevard Jean Moulin, 13385 Marseille Cedex 05, France; 2 Geneva University Hospitals and University of Geneva, Central Lab of Bacteriology, and Genomic Research Lab, 24 rue Micheli-du-Crest, CH-1211 Geneva 14, Switzerland; 3 Département des Maladies Respiratoires, Centre de Ressources et de Compétences pour la Mucoviscidose Enfants (CRCM), Hôpital Timone, Marseille, France; 4 Département des Maladies Respiratoires, Centre de Ressources et de Compétences pour la Mucoviscidose Adultes (CRCM), Hôpital Sainte Marguerite, Marseille, France

## Abstract

**Background:**

*Staphylococcus aureus *is a major human pathogen responsible for a variety of nosocomial and community-acquired infections. Recent reports show that the prevalence of Methicillin-Resistant *S. aureus *(MRSA) infections in cystic fibrosis (CF) patients is increasing. In 2006 in Marseille, France, we have detected an atypical MRSA strain with a specific antibiotic susceptibility profile and a unique growth phenotype. Because of the clinical importance of the spread of such strain among CF patients we decided to sequence the genome of one representative isolate (strain CF-Marseille) to compare this to the published genome sequences. We also conducted a retrospective epidemiological analysis on all *S. aureus *isolated from 2002 to 2007 in CF patients from our institution.

**Results:**

CF-Marseille is multidrug resistant, has a hetero-Glycopeptide-Intermediate resistance *S. aureus *phenotype, grows on Cepacia agar with intense orange pigmentation and has a thickened cell wall. Phylogenetic analyses using Complete Genome Hybridization and Multi Locus VNTR Assay showed that CF-Marseille was closely related to strain Mu50, representing vancomycin-resistant *S. aureus*. Analysis of CF-Marseille shows a similar core genome to that of previously sequenced MRSA strains but with a different genomic organization due to the presence of specific mobile genetic elements i.e. a new SCCmec type IV mosaic cassette that has integrated the pUB110 plasmid, and a new phage closely related to phiETA3. Moreover this phage could be seen by electron microscopy when mobilized with several antibiotics commonly used in CF patients including, tobramycin, ciprofloxacin, cotrimoxazole, or imipenem. Phylogenetic analysis of phenotypically similar h-GISA in our study also suggests that CF patients are colonized by polyclonal populations of MRSA that represents an incredible reservoir for lateral gene transfer.

**Conclusion:**

In conclusion, we demonstrated the emergence and spreading of a new isolate of MRSA in CF patients in Marseille, France, that has probably been selected in the airways by antibiotic pressure. Antibiotic-mediated phage induction may result in high-frequency transfer and the unintended consequence of promoting the spread of virulence and/or antibiotic resistance determinants. The emergence of well-adapted MRSA is worrying in such population chronically colonized and receiving many antibiotics and represents a model for emergence of uncontrollable super bugs in a specific niche.

**Reviewers:**

This article was reviewed by Eric Bapteste, Pierre Pontarotti, and Igor Zhulin. For the full reviews, please go to the Reviewers' comments section.

## Background

*Staphylococcus aureus *is a major human pathogen responsible for a variety of nosocomial and community-acquired infections ranging from mild to life -threatening diseases [1]. Along with the spread of this bacterium, an increase of antibiotic resistance has been reported over the last decades. Since the early sixties, methicillin-resistant *S. aureus *(MRSA) has caused large, life-threatening nosocomial outbreaks worldwide [2]. Initially, early isolates were also resistant to others antibiotics, and gentamicin-resistant MRSA (GR-MRSA) became epidemic in Australia, the United States, and Europe in the 1980s [3]. During the past two decades, the prevalence of MRSA involving both nosocomial and community-acquired infections has increased throughout the world [4]. In the late 1990s, community-acquired MRSA (CA-MRSA) showing specific genomic determinants became of major concern worldwide [5]. The emergence and spread of new MRSA strains susceptible to gentamicin (GS-MRSA) has been reported over the last ten years in European countries, especially in France [6-8]. Recent advances in the field of genome sequencing have provided new insights into the genetic diversity of these pathogens [9,10] and enabled the development of parallel tools to study clinical isolates at the organism scale [11-13]. To date, thirteen fully annotated *S. aureus *genomes are publicly available with eight being published [11-16]. The genome sequences of *S. aureus *have shown a well conserved core region corresponding to approximately 80% of the genome, but also displays a wide diversity of accessory genetic elements [13]. These observations confirm important genetic diversity and high plasticity of the bacterium and suggest that these contribute to its adaptation to environmental changes, including antibiotic selection pressure.

Cystic fibrosis (CF) remains an important hereditary disease in Europe and is characterized by chronic suppurative airway disease with progressive respiratory insufficiency [17]. The CF airways may represent a model of emergence of resistant bacteria in this specific niche, where many different bacteria are in close contact, increasing the frequency of potential lateral gene transfer. About 50 to 80% of CF children and adolescents are chronically colonized or infected by *Staphylococcus aureus *and are regularly treated with antibiotics without reaching complete eradication [17,18]. Such suboptimal antibiotic pressure in a selected niche is known to contribute to alter ecology in the environment and affect evolutionary trajectories especially for rapid evolution and artificial selection of multidrug resistant bacteria [19,20]. A recent report shows that the prevalence of MRSA infections in CF patients is increasing [18], a phenomenon attributed to the antibiotic selection pressure [21,22]. During chronic infection in CF, strong selective pressure is exerted on bacterial pathogens such as *Pseudomonas aeruginosa *and *S. aureus*, especially during treatment with tobramycin, ciprofloxacin and colistin, leading to discernable variations in the clonal lineages [23]. Phage mobilization contributes significantly to genome alteration in *S. aureus *during infection [24] and recent evidence has demonstrated that antibiotics such as ciprofloxacin and trimethoprim can cause phage induction in *S. aureus *isolates from CF patients [25]. Moreover, it is well known that coevolution with bacteriophages is a major factor for the evolution and diversification of bacterial populations that could lead to antibiotic resistance [26,27]. In this particular scenario, highly adapted *S. aureus *strains to a specific environment may emerge and spread among this sensitive patient population.

### Study design

**Figure 1:**
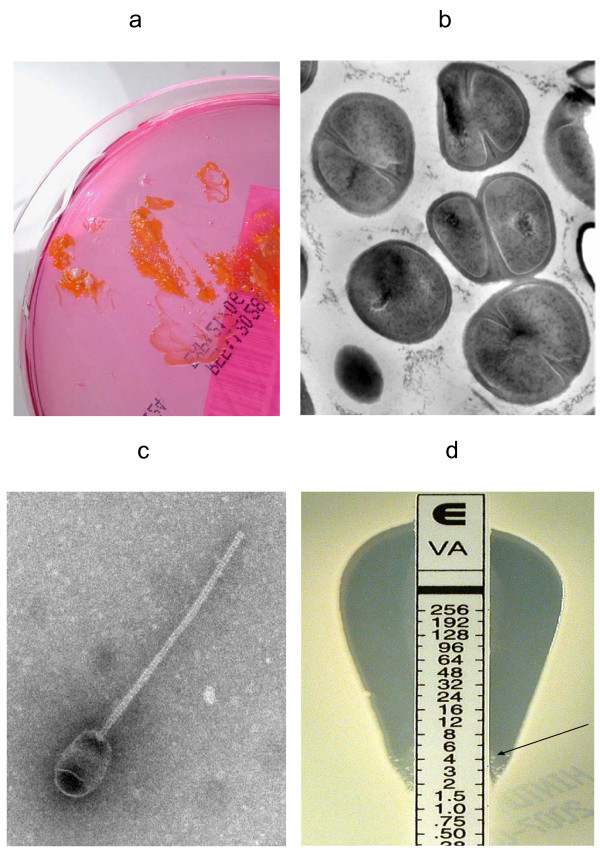
**Growth of GS-MRSA strain CF-Marseille on Cepacia agar showing intense orange pigmentation (**a**) and Transmission Electron Microscopy showing the cell wall thickness and abnormalities of septation (**b**)**. Strain CF-Marseille phage induced by antibiotics as viewed using electron microscopy (**c**) magnification (×140.000). Etest strips with vancomycin showing satellites colonies growing in the inhibition region (**d**).

In 2006 in Marseille, France, during an epidemiological survey of *S. aureus *in CF patients, we have detected an atypical GS-MRSA strain with a specific antibiotic susceptibility profile and a unique growth phenotype. The strain was susceptible to gentamicin and resistant to tobramycin, kanamycin, erythromycin, lincomycin, and ofloxacin. The isolate had a hetero-Glycopeptide-Intermediate phenotype of resistance (GISA) and grew with atypical intense orange pigmentation on Cepacia agar (Figure 1a). Because of the clinical importance of the spread of such strain among CF patients we sequenced the genome of one representative isolate (CF-Marseille) and compared this to the published genome sequences, to detect new genetic features responsible for pathogenicity, epidemicity or antibiotic resistance. For this purpose we used high throughput sequencing system (454 Life Science Corp., Roche) [28] coupled with microarrays and molecular genotyping methods to decipher specificities of this isolate in the CF population. We also conducted a retrospective epidemiological analysis on all *S. aureus *isolated from 2002 to 2007 in CF patients from our institution to understand dynamics of change and spread of the different strain phenotypes. Finally, specific sequences found in the newly sequenced genome were used to design primers to trace the strain in an epidemic setting [29].

## Results

### Phenotype of strain CF-Marseille

The cell wall of CF-Marseille was significantly thicker (33.5 +/- 5.8 nm, n = 100 measurements) than MSSA strain CIP 76.25 (24.7 +/- 4.0 nm, n = 100 measurements) (p < 10^-3^) (Figure 1b). Presence of phages was not visualized by electron microscopy without induction whereas phages were seen when antibiotics were added to the medium (Figure 1c). Antibiotics able to induce phages were fusidic acid, tobramycin, ciprofloxacin, cotrimoxazole, erythromycin, rifampin and imipenem whereas oxacillin, ceftazidime, vancomycin, fosfomycin, thiamphenicol, and colistin were not. Apart from its specific antibiotic susceptibility profile, CF-Marseille has a vancomycin MIC of 2–2.5 μg/ml (Etest strips with cell suspensions calibrated at 2 McFarland units) but satellite colonies grew within the ellipse of growth inhibition (Figure 1d). Profile analysis population with teicoplanin confirmed that some colonies were able to grow at 4 μg/ml (Additional file 1), thus displaying a hetero-GISA phenotype [30].

### CF-Marseille Genome and specific transcription profiling

The 454 sequencing of GS-MRSA strain CF-Marseille genome gave a total of 19,064,566 nucleotides that gave a total of 131 assembled contigs with sizes ranging from 546 to 210,978 bp. The contig sizes sum up to 2,829,971 bp. The total contigs size of 2.83 Mbp provides a lower bound of the chromosome size of CF-Marseille. The 131 contigs are available in the EMBL Nucleotide Sequence Database, accession number DS:71627. We compared the CF-Marseille strain to other *S. aureus *strains on the basis of their protein contents. A CoDing Sequence (CDS) prediction was performed with the Glimmer software 1, which revealed the presence of 2736 hypothetical proteins in the 131 contigs. As compared to the other *S. aureus *proteins available in the UniProtKB/Swiss-Prot database 2 (considered strains were N315, MW2, USA300, Mu50, NCTC 8325, COL and MRSA252), 2675 out of 2736 predicted proteins showed a match of at least 90% of aminoacid identity. The strains USA300/Mu50 shows the closest protein content with 2522 matches, corresponding to more that 95% of the detected CF-Marseille proteome. The 61 proteins that did not match mostly consist of phagic proteins and very short CDS that are likely to be false positive (Open reading frame that do not correspond to CDS).

Analysis of the accessory genome of CF-Marseille revealed the presence of a new phage of 44 Kb closely related to the recently sequenced phage phiETA3 found in *S. aureus *strain JH1-JH9 [31]. Similarity search at the protein level showed that parts of this element are found in numerous other phages including phiETA3, phiROSA, phiNM4 or phage 96, whereas the function of an important number of putative ORFs remain hypothetical.

Most of the genes differentially expressed between strains found in CF patient and isolates never found in CF patient (see Additional file 2), involved phage elements or resistance determinants, which is consistent with the CGH results. The only gene showing higher expression in the non-CF isolates compared to CF isolates was *spxA*, a transcription regulator involved in the biosysnthesis of thioredoxin reductase, an enzyme found down-regulated during treatment with hypochlorite [32] or in the presence of berberine chloride [33] and whose deletion yields to the accumulation of biofilm onto surface. Comparison between strains currently isolated in Marseille and CF-Marseille yields a limited number of differentially regulated genes. All these genes belong to phage elements and resistance determinants. Among these were *ermC *(a gene conferring resistance to erythromycin) and ORFs of the phage (Additional file 2). Most of the targets showing differential expression corresponded to up-regulated genes in one of the phages identified in strain CF-Marseille and potentially in other strains of our collection.

### Antibiotic resistance gene s in the genome of strain CF-Marseille

**Figure 2:**
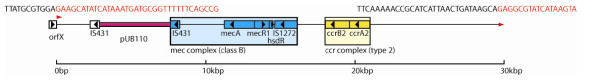
**Schematic representation of the SCC*mec *element of CF-Marseille (Hx1203407144) showing combined structure built of part of SCC*mec *II and IV elements**.

In the genome of CF-Marseille, resistance to beta-lactams and ofloxacin are chromosomally-encoded (Table 1). Indeed, resistance to ofloxacin can be attributed to a chromosomal mutation that results in an aminoacid change Ser84Leu in the gyrase gene *gyrA *(contig 00393). The SCC*mec *element in CF-Marseille is novel. Although sequence analysis suggested that it is a type IV cassette containing recombinases *ccrA2 *and *ccrB2*, genotyping experiments showed relatedness with N315, a HA-MRSA containing a type II cassette (accession number AM943017). A refined analysis of recombinase sequences revealed that *ccrA2 *of CF-Marseille was identical to *ccrA2 *of strain N315 whereas *ccrB2 *of CF-Marseille was similar to that of strain MW2. This cassette was also peculiar with a size of 29493 bp with the integration of the pUB110 plasmid carrying kanamycin and bleomycin resistance (Figure 2). Thus the SCC*mec *cassette of CF-Marseille is a mosaic of elements from a type II (nosocomial origin) and a type IV (community origin) cassette. Resistance to macrolides (erythromycin) and tetracycline is encoded on plasmid pWBG738 (contig 00364, 98% homology with Genbank sequence 007209) identified in CA-MRSA strain ST1-MRSA-IV similar to strain MW2. Other antibiotic encoding genes were found in the genome including a metallo-betalactamase (contig 00393 and 00379) as well as several ABC transporters.

**Table 1:** Resistance determinants in CF-Marseille.

Antibiotic Resistance	Gene	Origin of genes
Beta-lactams	*mec*A metallobetalactamase	SCC*mec *type IV
Fluoroquinolones	*gyrA *(Ser83Leu)	DNA gyrase
Kanamycin -	aadD	pUB110 on SCC*mec*
Tobramycin		
Bleomycin	bleO	pUB110 on SCC*mec*
Erythromycin	*ermC*	Plasmid
Tetracycline	*tet*	Plasmid
Efflux pumps	ABC transporters	

### Origin and spreading of CF-Marseille

**Figure 3:**
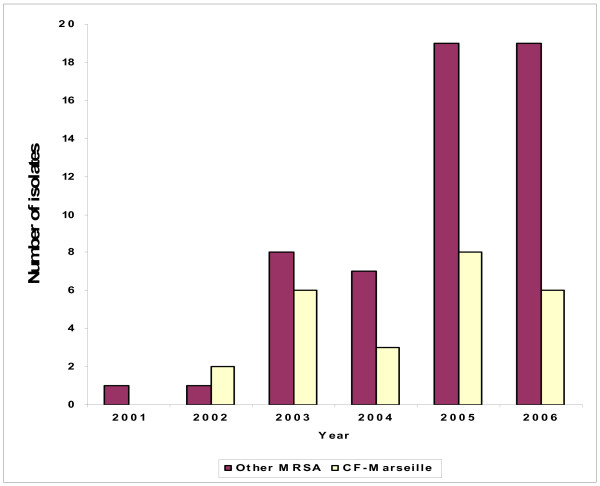
**Repartition of CF-Marseille phenotype among MRSA per year**.

**Figure 4:**
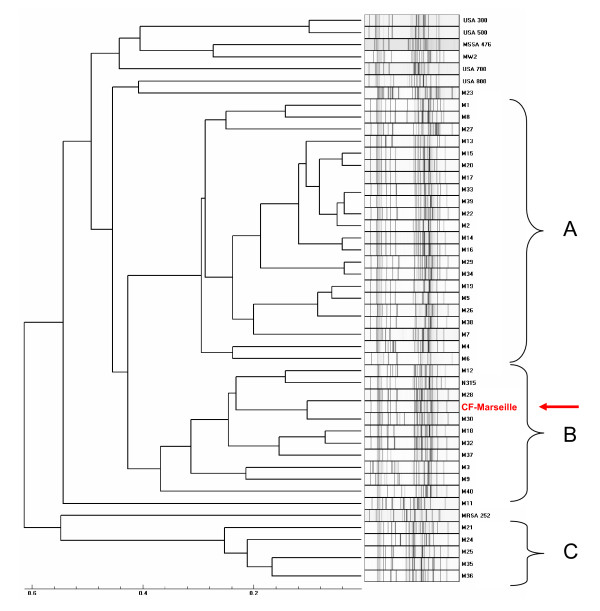
**Results of the MLVA analysis on DNAs from *S. aureus *strains with the respective pattern on the right and the computed dendrogram on the left**. Strains are designed by their number (Additional file 3). Group A is formed exclusively by MRSA isolated both from CF and non-CF patients, group B is composed of both MRSA and MSSA strains, isolated from CF and non-CF patients and group C is exclusively formed by MSSA strains isolated from CF patients. Arrow shows CF-Marseille.

From May 2001 to December 2006, 108 adults (age ≤ 18 years) and 98 children or adolescents (age < 18 years) were followed in the two CF centres. Overall, 127 patients (61.7%) were found to be colonized at least once by *S. aureus *(82 children, 83.7% and 45 adults, 41.7%) and represented 699 isolates encompassing 270 non redundant strains (182 in children, 67.4% and 88 in adults, 32.6%). Among these 270 strains, 80 were resistant to methicillin (29.6%) with 56 (70%) in children and 24 (30%) in adults. Among these 80 MRSA isolates, 18/56 (32.1%) and 7/24 (29.2%) showed the CF-Marseille phenotype in children and adults, respectively. The CF-Marseille phenotype was firstly detected in 2002 and since then newly detected isolates have increased to reach a total of 25 patients (Figure 3). Presence of CF-Marseille was significantly associated with *P. aeruginosa *colonization (22/25 patients) when compared to patients colonized with MSSA (56/190) (p < 0.05). In addition, when compared to acquisition of MSSA, the risk of acquisition of CF-Marseille was twice higher in patients with *P. aeruginosa *infection and colistin treatment (Risk ratio = 2.00 [1.02–3.99]; p = 0.047). Phylogenetic relationship between the 29 additional strains isolated in the same hospital from CF patients and from 10 non-CF patients using multi-locus variable number of tandem repeat assay (MLVA) is given in Figure 4. Patient information and typing of the strains are given in Additional file 3. By combining these results with those of oxacillin/methicillin sensitivity, it can be seen that one large group is formed exclusively of MRSA isolated both from cystic fibrosis and non-CF patients (group A). This cluster is composed of SCC*mec *IV isolates with a type 1 *agr *locus. The second group is composed of both MRSA and MSSA strains, isolated from cystic fibrosis and non-CF patients (group B). CF-Marseille belongs to Group B (arrow on Figure 4). In this subgroup, strain CF-Marseille co-clustered with several isolates harbouring SCC*mec *IV element and type 2 *agr *loci but also with strains harbouring SCC*mec *II elements. Based on the structure of the MLVA tree, this cluster segregated distantly from control community acquired isolates such as MW2 or USA300. Among the sequenced isolates used as controls for the MLVA assay, strain CF-Marseille appears highly related to N315, a Japanese isolate harboring a type II SCC*mec *element showed to evolve to the GISA phenotype. A third smaller group (Group C) is exclusively formed of MSSA strains isolated from cystic fibrosis patients and it is separate from Group A and Group B in the dendrogram analysis. This small cluster of isolates harbours *agr *3 locus and the TSST-1 gene. Finally, 14 out of 21 MRSA strains from CF patients and 3 out of 10 MRSA strains from non-CF patients contained the phage found in CF-Marseille as determined using specific primers and probe. Complete genome hybridization shows that CF-Marseille appears clearly related to N315/Mu50 in terms of genomic content, as shown by microarray CGH experiments, covering 100% and 95% of N315 and Mu50 ORFs, respectively as well as 96% of ORFs detected in 6 other sequenced strains (Additional file 4). In terms of genetic content, the genome of CF-Marseille displays some alterations compared to other strains currently isolated in Marseille. Plasmids and other mobile elements encoding for specific resistance determinants were clearly visible during this comparison. Parts of sequenced phages were also present in CF-Marseille and absent in the other strains (in particular segments of a phage characterized in USA300). CF-Marseille was also the only strain to harbor enterotoxin m and the *sdrD *genes. In addition, some important genes such as part of the *agr *locus (particularly the conserved gene *agrB*) presented some divergence between the 2 populations. List of genes absent in CF-Marseille but present in the other isolates contained only mobile genetic elements such as resistance determinants or integrases originating probably from different phages harbored in the 2 strain populations.

## Discussion

In this study, we report the detection and increase in carriage of a unique MRSA strain in CF patients in Marseille, France. Retrospective epidemiological analysis during a 6 years period demonstrates that the prevalence of MRSA in our CF population was higher than previous reports worldwide with a rapid spreading during the last 2 years. In the United States, approximately 12% of CF patients have had infection with MRSA [34], although its prevalence may vary among centres [18]. In France, the "Observatoire National de la Mucoviscidose" in 1999 reported a prevalence of 57.6% of patients with *S. aureus *with 9.2% being MRSA. This percentage increased in 2004, with a mean of 14.5%, with similar results in children and adults (unpublished data). Overall in our two centres in Marseille, prevalence of *S. aureus *was 61% with a percentage of MRSA of approximately 30%, with the CF-Marseille phenotype representing more than 30% of the MRSA. In an outbreak of resistant bacteria, the identification of genes associated with virulence or antibiotic resistance should be rapidly performed [35]. For this purpose, we have used the most advanced and rapid approach to characterize this strain at the organism scale. The use of high-throughput sequencing engineered by 454 Life Science Corp. gives approximately 100-fold increase in throughput over current Sanger sequencing technology with 96% coverage and 99.96% accuracy in only 4 hours [28]. The short lengths of sequences obtained (approximately 100 bp) makes the presence of repeated regions above this size an obstacle to the molecular closing of the genome. In our case, because isolates of the same species were already sequenced and as only gene content appears relevant, we speculate that repeated regions may not be a constraint for the detection of important genes at the molecular level without finishing the assembly of the genome [36].

Analysis of CF-Marseille shows a similar core genome to that of previously sequenced MRSA strains but with a different genomic organization due to the presence of specific mobile genetic elements i.e. a new SCC*mec *type IV cassette that has integrated the pUB110 plasmid, and a new phage closely related to phiETA3 [31]. Interestingly, the 454 technology was more powerful and complementary to the microarray technology since these two genetic elements were not elucidated using only microarray analysis. Comparison of CF-Marseille strain with other available genomes using CGH and MLVA showed that CF-Marseille was closely related to strain Mu50 [12], representing vancomycin-resistant *S. aureus*. This observation was confirmed by MLST, as strain CF-Marseille co-clustered with different strains previously described as GISA, such as ST5, ST239 or ST105. Phylogenetic analyses indicate that strain CF-Marseille probably has a great potential to develop a GISA phenotype. Phenotypically, CF-Marseille appears susceptible to vancomycin (MIC = 2 μg/ml) but exhibits low level resistance with subpopulations being able to grow at 4 μg/ml with a thickened cell wall similar to that of strain Mu3, the quintessential hetero-GISA [30,37]. This phenotype has been recently reported in CF patients in a Belgian hospital [38]. The mechanism of glycopeptide resistance in *S. aureus *is not fully understood, although changes in cell surface phenotypes have been described including a thickened cell wall, an increased amount of glutamine non-amidated muropeptides and decreased cross-linking of the cell wall peptidoglycan [39,40]. The intense orange pigmentation may also be linked to a h-GISA phenotype since it has been shown that *S. aureus *under stress conditions may activate *sigB *regulon, associated with the overexpression of carotenoïde synthesis and an increase in cell wall thickness and glycopeptide resistance [41,42].

Molecular detection, using primers and probe, of the new phage in additional strains isolated from CF patients was also possible thanks to the genome analysis and allowed us to design a specific tool to trace the epidemic strain in our institution. Interestingly, we found that MRSA isolates from non-CF patients may contain this new phage, suggesting that it could spread in our hospital as well. However, a striking difference was observed in strain CF-Marseille in that it shows a clear up-regulation of parts of phage genes as compared to all other strain groups tested by genome-wide transcription profiling. This observation appears important in the context of an infection as prophages have been shown to contribute to strain virulence through tissue tropism [31]. Because prophages are induced by stress conditions and antibiotic pressure [25,43], both conditions present in the airway of CF patients, our results clearly indicate that such spreading occurs in our hospital and between CF patients. It has been demonstrated that strong selective pressure on a bacteria and its phage during coevolution lead to the emergence of specific bacterial populations specialized in a selective environment [27].

In conclusion, we demonstrated the emergence and spreading of a new isolate of MRSA in CF patients in Marseille, France, that has probably been selected in the airways by antibiotic pressure. Our findings support the hypothesis that the dominance of specific multidrug-resistant *S. aureus *clones result from both the antimicrobial selective pressure and, as recently suggested, the dynamic association of different factors involved during the co-evolution of bacteria and host [44]. The increased level of antibiotic resistance and the emergence of such strains highlight the plasticity of *S. aureus *genomes and the remarkable speed of bacterial evolution, especially by horizontal gene transfer including bacteriophages, *S. aureus *pathogenicity islands, SCC*mec*, plasmids and transposons, to allow the bacteria to very rapidly adapt to a specific niche. The phages in *S. aureus *are a remarkable source of untapped genetic diversity [45] with more than 60% of predicted protein-encoded ORFs that cannot be annotated for structure or function [46]. Phylogenetic analysis of phenotypically similar h-GISA in our study also suggests that CF patients are colonized by polyclonal populations of MRSA that represents an incredible reservoir for lateral gene transfer and emergence of uncontrollable super bugs, as recently exemplified in two CF patients infected with MRSA carrying Panton-Valentine leukocidin toxin [47]. Antibiotic-mediated phage induction may result in replication and high-frequency transfer and the unintended consequence of promoting the spread of virulence and/or antibiotic resistance determinants as recently demonstrated with ciprofloxacin and beta-lactams and *S. aureus *[25,48] and ciprofloxacin and *P. aeruginosa *[49]. The speed with which resistance and virulence genes move between strains by lateral gene transfer in *S. aureus *is clinically worrisome in patients chronically colonized and receiving many antibiotics and represents a model for emergence of uncontrollable super bugs in a specific niche. We believe that particular effort should be initiated to make CF patients MRSA-free as soon as MRSA is detected to avoid the possibility of lateral gene transfer by generalized transduction induced by the use of antibiotics. The epidemiology of MRSA in CF patients from other centres and other countries should be examined and compared to identify potential reservoirs of particular strains as well as Vancomycin-Resistant *S. aureus *that could emerge in this population.

## Conclusion

In conclusion, we demonstrated the emergence and spreading of a new isolate of MRSA in CF patients in Marseille, France, that has probably been selected in the airways by antibiotic pressure. Genome analysis of this atypical MRSA using high throughput sequencing method and phylogenetic analyses revealed the presence of a new antibiotic inducible phage and a hGISA phenotype. Antibiotic-mediated phage induction may result in high-frequency transfer and the unintended consequence of promoting the spread of virulence and/or antibiotic resistance determinants. The emergence of well-adapted MRSA is worrying in such population chronically colonized and receiving many antibiotics and represents a model for rapid evolution and emergence of uncontrollable super bugs in a specific niche.

## Methods

### Epidemiology of CF-Marseille

All CF patients followed in the two CF reference centres at Marseille, France, were included in this study from May 2001 to December 2006 for the epidemiological analysis. Statistical analyses were performed using Epi Info 6.0 Software. *S. aureus *strains isolated from sputum samples were collected from January 2006 to December 2006 using routine laboratory culture methods and standard identification methods [50,51]. Criteria for selecting epidemic MRSA strains were susceptibility to gentamicin combined to resistance to oxacillin, tobramycin, and kanamycin. Twenty one of these isolates as well as 9 MSSA from CF patients and ten MRSA isolated in the same hospital during the same period in non-CF patients were further analyzed using genotypic markers and microarray experiments by using previously described procedures [52-54]. This study has been approved by our local ethic committee. No written consent was needed for this work in accordance with the "LOI n° 2004-800 relative à la bioéthique" published in the "Journal Officiel de la République Française" the 6 August 2004 since no additional sample was taken for the study.

### Phenotypic analysis of the epidemic clone

MRSA strain CF-Marseille, the prototype of GS-MRSA recovered in CF patient, was isolated in January 2006 from the sputum of a 14-year old CF girl. MIC values of antimicrobials were determined according to the Committee for Antimicrobial Testing of the French Society for Microbiology using a Vitek2* system (bioMérieux, Marcy l'Etoile, France) with Gram positive susceptibility test cards. MIC against vancomycin was tested using Etest strip (AB Biodisk, Solna, Sweden) performed at 0.5 and 2.0 McFarland inocula on BHIA as previously described [55]. Plates were incubated at 37°C and read after 48 h. CF-Marseille was also tested for glycopeptide-intermediate susceptibility by population analysis [37,55]. Finally, one hundred microliters of a bacterial suspension adjusted to McFarland standard 2.0 was spread on brain heart infusion agar (Becton Dickinson, Le Pont de Claix, France) plates with 6 mg/l of vancomycin (Merck, Lyon, France) as described previously [37,55]. Plates were incubated and growth observed after 48 h. MRSA strain CF-Marseille and vancomycin susceptible *S. aureus *(VSSA, strain CIP 7625) were examined with a transmission electron microscope Philips -Morgagni 368D (Fei Company, Eindhoven, The Netherlands). The strains were grown on trypticase soya agar at 37°C and were then stained with ruthenium red as described by Luft [56] prior to processing for electron microscopic examination. The cell wall thickness was observed using a Mega View II camera and measured using Analysis 3.2 and Soft Imaging System software. Statistical analysis was done using Epi Info version 6.0 software (CDC, Atlanta, Ga.); p values < 0.05 were considered statistically significant.

### Phage induction

CF-Marseille was grown for 2 hours in Trypticase soya broth (TSB, BioMérieux, Marcy l'Etoile, France) at 37°C. Mitomycin C (SIGMA-ALDRICH, Saint-Quentin Fallavier, France) was used in phage mobilization as described previously [31]. Briefly, 1 μg/ml of mitomycin C was added to the TSB culture and after 3 hours of incubation with shaking at 30°C, the cell lysate was passed through 0.22 μm filters. Plaque assay was performed to verify phage induction using *S. aureus *strain CIP 76.25. The effects of sub-inhibitory concentrations of other antibiotics on phage induction of CF-Marseille were also analyzed. The following antibiotics were used as described above: oxacillin (PANPHARMA, France) (8 μg/ml), ceftazidime (GlaxoSmithKline, Marly-Le-Roi, France) (8 μg/ml), imipenem (Merck Sharp & Dohme-Chibret, Paris, France) (10 μg/ml), tobramycin (MERCK, Lyon, France) (8 μg/ml), ciprofloxacin (MERCK, Lyon, France) (8 μg/ml), gentamicin (PANPHARMA, France) (0.5 μg/ml), rifampicin (Sanofi aventis, Paris, France) (0.5 μg/ml), vancomycin (MERCK génériques, Lyon, France) (1 μg/ml), fusidic acid (LEO, St; Quentin Yvelines, France) (0.5 μg/ml), fosfomycin (ERN, S.A., Barelona, Espain) (8 μg/ml), thiamphenicol (Sanofi aventis, Paris, France) (10 μg/ml), sulfamethoxazole-trimethoprim (Roche) (10 μg/ml), erythromycin (AMDIPHARMA, Dublin, Irlande) (8 μg/ml), metronidazole (Fresenius Kabi, Sèvres, France) (10 μg/ml), and colimycin (Sanofi Aventis, Paris, France) (300 IU/ml). Presence of induced phages was examined with a transmission electron microscope Philips -Morgagni 368D as described above.

### Genome analysis of CF-Marseille

#### Sequencing and assembly of CF-Marseille

*S. aureus *strain CF-Marseille was grown on trypticase soya agar, then harvested and suspended in TE buffer. DNA was extracted according the classical lytic treatment using SDS and proteinase K followed by phenol-chloroform isoamyl alcohol extraction. DNA was solubilized in TE buffer and visualized on agarose gel stained by ethidium bromide. Genome sequencing was performed under previously described conditions using the 454 technology (454 Life Sciences, Branford, USA) [28]. Assembly was performed using Newbler software of the 454 suite package. Mapping was performed using Projector 2 tool with or without masking repeats [57] and compared to contig alignment using NUCmer of MUMmer 3.0 program [58]. The contigs which did not automatically map by Projector program but had significant matches with reference genomes were mapped using NUCmer tool. The contigs which did not map using both tools were subjected to further BLAST analysis [59] against *S. aureus *genomes. The unmatched contigs with available *S. aureus *genomes were also subjected to BLAST search (E-value = 10-4) against the non-redundant GenBank database to identify their homologs with genomic sequences of other genomes.

#### Design of primers and probe to target the phage related sequences

Primers and Taqman* probe used to target the phage related sequences were 00394F (5'-AAATGGCTTGGAGGAATTGAAC-3') and 00394R (5'-ACCAAATGCAACACAACGAATG-3') and 00394probe (6FAM- TGGGAACCTAGTGGCAGATCCAGA-TAMRA) that yield a 182 bp sequence.

#### Genotyping of representative isolates

Genomic DNA of the 40 isolates described above were extracted from one colony as previously described [60]. All qPCR tests were performed with oligonucleotides designed using PrimerExpress (PE Biosystems, Foster City, CA, USA). Typing of I to IV SCC*mec *cassette elements and of *agr*-group were performed using previously published methods [60,61]. Presence of phage, PVL, TSST-1 and exfoliatin toxins was assessed using specific oligonucleotides. Conditions and settings used for these analyses were previously described [60,61]. Multi Locus sequence Typing (MLST) was performed using previously described procedures and primers [52]. Allele numbers were assigned according to the program available from the MLST Web site . Multiple-locus VNTR assay (MLVA) typing assay was performed as previously described [53,54] using 9 pairs of primers targeting VNTR containing genes and one additional pair or primers targeting the *mecA *gene. Main clusters of strains were identified using previously described analytical settings [53]. Representative isolates in these main clusters of strains were selected for microarray experiments.

### Microarray design

The microarray was manufactured by in situ synthesis of 10'807 long oligonucleotide probes (Agilent, Palo Alto, CA, USA), selected as previously described [62]. It covers >98% of all ORFs annotated in strains N315 and Mu50 [12], MW2 [11] and COL [15], NCTC8325, USA300 [14], MRSA252 an MSSA476 [13] including their respective plasmids. Genomic DNA (gDNA) was prepared from isolated colonies grown overnight on Mueller Hinton (MH) agar at 37°C. Briefly: 10^9 ^cells were lyzed in 100 μLTris EDTA buffer (10 mM Tris-1 mM EDTA, pH = 8) containing 50 μg/ml lysostaphin (Ambicin from Applied Microbiology, Tarrytown, NY) for 10 min at 37°C. DNA was then isolated and purified using DNeasy^® ^kit (Qiagen, Hilden, Germany) according to manufacturer's instructions, including RNAse treatment. DNA quantification and protein contaminations were assessed by using a NanoDrop^® ^ND-1000 Spectrophotometer (NanoDrop Technologies, Inc. Rockland, DE).

#### Microarray complete genome hybridization (CGH) and scanning

Test and reference gDNAs (1 μg) were labelled with cyanine 3 or cyanine 5 dCTP (NEN, Perkin Elmer, Foster City, USA) using the BioPrime DNA Labelling kit (Invitrogen, Carlsbad, CA) following manufacturer's instructions. Unincorporated fluorescent nucleotides were removed using Centrisep columns (Princeton separations, EMP Biotech, Berlin, Germany). Cy 3 labelled gDNAs from the four reference strains used to design the microarray (0.125 μg from each strain) were mixed with 0.5 μg of Cy 5 labelled test gDNA in hybridization buffer (Agilent Technologies, CA, USA), to a total volume of 250 μl. Hybridization mixture was heated to 95°C for 2 minutes and then hybridization was performed for 17 hours at 60°C with rotation in a dedicated hybridization oven (Robbins Scientific, Sunnyvale, CA, USA). Stringent washings were then performed according to manufacturer's instructions. Slides were dried under nitrogen flow, and scanned (Agilent Technologies, CA, USA) using 100% Photon Multiplier Tube (PMT) power for both wavelengths using a Agilent scanner.

#### Expression microarrays

Preparation of labelled nucleic acids *S. aureus *strains were grown in Muller-Hinton broth for 4 hours. Total RNA was extracted from 2 mL of cells at 2–3 × 10^9 ^cells/ml, using the RNeasy kit (Qiagen, Basel, Switzerland), as previously described (15;16). After additional DNase treatment, the absence of remaining DNA traces was evaluated by quantitative PCR (SDS 7700; Applied Biosystems, Framingham, MA) with assays specific for 16s rRNA [63,64]. Batches of 10 μg total *S. aureus *RNA were labelled by Cy-3dCTP using the SuperScript II (Invitrogen, Basel, Switzerland) following manufacturer's instructions. Labelled products were then purified onto QiaQuick columns (Qiagen). Purified genomic DNA from the 8 sequenced strains was extracted (DNeasy, Qiagen), labeled with Cy-5 dCTP using the Klenow fragment of DNA polymerase I (BioPrime, Invitrogen, Carlsbad, CA) [54]. Cy5-labeled DNA (0.125 μg per stain) and Cy3-labeled cDNA (10 μg) mixture was diluted in 250 μl Agilent hybridization buffer, and hybridized at a temperature of 60°C for 17 hours in a dedicated hybridization oven (Robbins Scientific, Sunnyvale, CA, USA). Slides were washed, dried under nitrogen flow, and scanned (Agilent, Palo Alto, CA, USA) using 100% PMT power for both wavelengths. Data were extracted and processed using Feature Extraction™ software (version 8, Agilent).

#### Microarray analysis

Fluorescence intensities were extracted using the Feature extraction software (Agilent, version 8). Local background subtracted signals were corrected for unequal dye incorporation or unequal load of the labelled product. For CGH analysis experiments, the algorithm consisted of a rank consistency filter and a curve fit using the default LOWESS (locally weighted linear regression) method. Additional software was developed in house to analyze the processed data. This software filtered the data to exclude irrelevant values, as flagged by the extraction software. Background noise of each experiment was evaluated by computing the standard deviation of negative control intensities. Features whose intensities were smaller than the standard deviation value of the negative controls were considered as inefficient hybridization and discarded from further analysis. The software calculated for each spot the logarithm of the ratio between the test channel and the control channel (log ratio). Since the control signal is present in each spot, this log ratio corresponds to a per feature normalization. Computed log ratio values were further sorted into 150 bin categories and fitted with a Gaussian distribution curve, using the Levenberg-Marquardt algorithm. The software estimated the presence probability of each oligonucleotide probe (EPP), as previously described [10,54]. As clearly documented in the work of Kim et al., we used the most stringent EPP value as our study was focused on "strict divergent gene analysis parameter" [65]. Values showing an EPP <1% (each oligonucleotide probe) were extracted and considered as absent features in the test channel. The list of absent features from each experiment was then clustered by the software using Dice distance and Group average linkage algorithm to construct a hierarchical cluster tree [66].

For expression analysis, local background-subtracted signals were corrected for unequal dye incorporation or unequal load of labelled product. The algorithm consisted of a rank consistency filter and a curve fit using the default LOWESS (locally weighted linear regression) method. Data consisting of three independent biological experiments were expressed as Log10 ratios and analyzed using GeneSpring7.0 (Silicon Genetics, Redwood City, CA, USA). Statistical significance of differentially expressed genes was identified by variance analysis (ANOVA) [63,67], performed using GeneSpring, including the Benjamini and Hochberg false discovery rate correction (5%).

### Statistical analysis

Statistical analysis were done using Epi Info Software version 6.0  for Chi-square – Mantel-Haenszel two-tailed test and risk ratio.

### Accession numbers

The 131 contigs of the genome of CF-Marseille are available in the EMBL Nucleotide Sequence Database under accession number DS:71627. The SCCmec element in CF-Marseille is available in the EMBL Nucleotide Sequence database under accession number AM943017.

## Abbreviations

CA-MRSA: Community-Acquired Methicillin-Resistant *Staphylococcus aureus*; CDS: CoDing Sequence; CF: Cystic Fibrosis; CGH: Complete Genome Hybridization; GISA: Glycopeptide-Intermediate *Staphylococcus aureus*; ORF: Open Reading Frame; GS-MRSA: Gentamicin Susceptible Methicillin-Resistant *Staphylococcus aureus*; MLST: Multi Locus Sequence Typing; MLVA: Multi Locus VNTR Assay; MRSA: Methicillin-Resistant *Staphylococcus aureus*; PVL: Panton Valentine Leukocidin; MSSA: Methicillin-Susceptible *Staphylococcus aureus*; TSST: Toxic Shock Syndrome Toxin; SCCmec: Staphylococcal Chromosomal Cassette *mec*; VSSA: Vancomycin-Susceptible *Staphylococcus aureus*.

## Competing interests

The authors declare that they have no competing interests.

## Authors' contributions

JMR was involved in the conception and design of the study, data analysis and interpretation, writing the article and was responsible for the manuscript. PF was involved in data analysis and interpretation of genotyping, microarray and genome sequencing and drafting the article. DH, GF and YM were invovled in data analysis, interpretation of genome sequencing and drafting the article. FB was involved in the acquisition and interpretation of data, culture and sequence, phage induction and drafting the article.HR was involved in data analysis and interpretation of epidemiology, statistical analysis, revision of the manuscript for important intellectual content. EB, NS, JCD, JS, MRG and SB were involved in patients sampling, epidemiological data analysis and interpretation. JS and DR were involved in conception and design of the study, revision of the manuscript for important intellectual content.

## Reviewers' comments

### Reviewer's report 1

*Eric Bapteste, Dalhousie University, Canada *

Rolain et al. submitted a solid and well-written scientific work. They certainly discovered that an atypical MRSA strain thriving in Marseille harbours an antibiotic inducible bacteriophage. Although this is definitely worth to be published, I am not sure however that this paper belongs to Biology Direct. The analyses presented here seem more interesting from a medical point of view than from an evolutionary perspective, or than from a perspective of comparative genomics. It is thus unclear to me why these authors did not submit their manuscript to the Lancet or another such review. In Biology Direct readers would likely expect deeper (comparative) genomic analyses of this newly sequenced strain of *Staphylococcus aureus*. For instance, in their conclusion, the authors comment about lateral gene transfer, the genomic plasticity and the conservation of core genes in their strain, but they do not present any phylogenomic analyses to back up their claims. As a result, their discussion seems a bit vague, while a deeper analysis could prove of further interest to biologists as well as to the medical community. In terms of genomics, lots of interesting discoveries remain to be done with their data.

Finally, the MLVA tree – and the methods behind it- should be better explained; a list of the abbreviations used should be introduced in the beginning of the paper to help the reader.

#### Author's response

*We appreciate the comments of reviewer's 1 and we believe that our analyses are both interesting from a medical point of view and from an evolutionary perspective, accessible to both scientists and clinicians. In fact this paper has been reviewed before Biology Direct in other journals (Genome Research, Nature Medicine and PLOS Medicine). Although comments were mainly favourable, specific concerns were either that it was a dubious assumption to speculate that it would not be necessary to close the genome or that the advance provided by these results were specific to this particular strain. To the best of our knowledge this genome sequence of a MRSA isolated in CF patients is the first to be reported worldwide. From an evolutionary point of view our results demonstrate the negative impact and rapid evolution of bacterial community in a specific niche exposed to many antibiotics for the selection of well-adapted multidrug resistant bacterial clones that could spread in the population. As suggested by reviewer we have added phylogenomic analyses as well as more comments and details about MLVA methods and results to go thoroughly into lateral gene transfer and genome plasticity in bacterial evolution in the discussion section. A list of abbreviations has been added*.

### Reviewer's report 2

*Pierre Pontarotti, Université d'Aix-Marseille, France *

Rolain et al. report the complete sequence of a representative isolate of a Methicillin-Resistant *Staphylococcus aureus *from a cystic fibrosis patient (name strain CF-Marseille) and have conducted a comparative genome analysis with the published sequences. This comparative analysis shows that the CF Marseille strain displays a similar core genome with other sequenced MRSA, with some apomorphism due to mobile elements and a new phage closely related to Phi ETA3. The bioinformatics and microbiologic analysis are robust and the paper is highly informative. I would, however, advise the author to write a short introduction on the origin of multi- antibiotic resistance and artificial selection (see for example Palumbi: Humans as the World's Greatest Evolutionary Force Science 7 September 2001: Vol. 293. no. 5536, pp. 1786 – 1790 science.293.5536.1786). This will be helpful for the BD readers that are not specialists in the domain. An explanation of the role of virus (that do not encode for resistance antibiotic genes) in the emergence of antibiotic resistance is also necessary, as this role is not well explained for a non specialist.

#### Author's response

*We thank reviewer's 2 for the comments of our article. As suggested we have added a short introduction on the origin of multidrug resistance and artificial selection as well as an explanation of the role of virus in the emergence of antibiotic resistance (see introduction and discussion sections, respectively). Indeed, the main problem in CF patients is that they are chronically colonized and treated with many antibiotics for years that facilitate the emergence of such 'super-adapted' bacteria that could spread in the population*.

### Reviewer's report 3

*Igor Zhulin, University of Tennessee, USA*

The paper by J.-M. Rolain et al. describes a genomic analysis of a multidrug-resistant strain of S. aureus. I am curious why the authors decided to send their study to Biology Direct: this work would be more appropriate for a journal dedicated to infectious diseases (e.g. Journal of Clinical Microbiology). I am not sure that clinicians who need to know about this would necessarily read Biology Direct. Nonetheless, this paper presents a very interesting case: a strain, which is resistant to multiple antibiotics, has emerged from cystic fibrosis patients and the mechanism for this emergence is phage induction by administered antibiotics. The phage activity is likely responsible for promoting the spread

This is a straightforward study from the genome analysis point of you. It does show the power of array-based pyrosequencing in obtaining critical biological data in a very short time. Clearly, in this particular case, there was no need to spend extra time and money to chase the remaining 4% of DNA and close the genome. It became obvious upon analysis of the genomic data that sequences unique to this strain (in comparison with a number of available genomes of various *S. aureus *isolates) comprise a novel phage and a modified mobile genetic element. The rest of the study links this genomic data to the biology of the pathogen.

Despite having a degree in microbiology, I am not quite qualified to comment on the epidemiological aspects of this work. However, I read the paper with a great interest and did not notice any obvious flaws in experimental design and interpretation.

Overall, this finding appears to be important from the medical perspective and will contribute to better understanding of infectious diseases.

#### Author's response

*We appreciate and we agree with the comments of reviewer's 3. In fact, as explained in reviewer's 1 responses, this paper has been reviewed before Biology Direct in other journals (Genome Research, Nature Medicine and PLOS Medicine). One major concern by reviewers was the fact that in our study the genome was not closed. However our results clearly prove that closing the genome was not necessary to decipher the genome content of the strain very rapidly since other available genomes could be used for comparisons and mapping. Moreover, the 454 technology and microarray assay proved to be complementary for analysis of this new strain. We believe that the importance of understanding specific mechanisms of drug resistance and emergence of such bacteria in the setting of Cystic Fibrosis will encourage clinicians to read this paper in Biology Direct from a medical point of view*.

## Supplementary Material

Additional file 1 **Figure S1. Profile analysis population with teicoplanin showing the h-GISA population.** Click here for file

Additional file 2 **Table S1. List of genes down- and up-regulated in CF-Marseille as compared to other available genomes**. Click here for file

Additional file 3 **Table S2. Patient information and typing of the strains. Study n°: number attributed to the strain in this study. R, resistant; S, sensitive. MecA: detection of the *mec*A gene**. SCC Mec: type of the SCC*mec *cassette; SCND, cassette type not determined (i.e. not 1, 2 or 4). AGR: *agr *type. PVL, TSST-1, EXFO-A, EXFO-B: detection of the gene coding for the toxin. Click here for file

Additional file 4 **Figure S2. Complete Genome Hybridization results with other strains.** Gene and strain clustering by GeneSpring (a). Dendogram based on the presence and absence of genes in the 12 strains on CGH results (b). Click here for file
