# Supplementary figures and images for: Genomic analysis of an emerging multiresistant Staphylococcus aureus strain rapidly spreading in cystic fibrosis patients revealed the presence of an antibiotic inducible bacteriophage
Source: Biol Direct. 2009 Jan 13;4:1. doi: 10.1186/1745-6150-4-1 (PMC2629466; doi:10.1186/1745-6150-4-1)

Figure S1

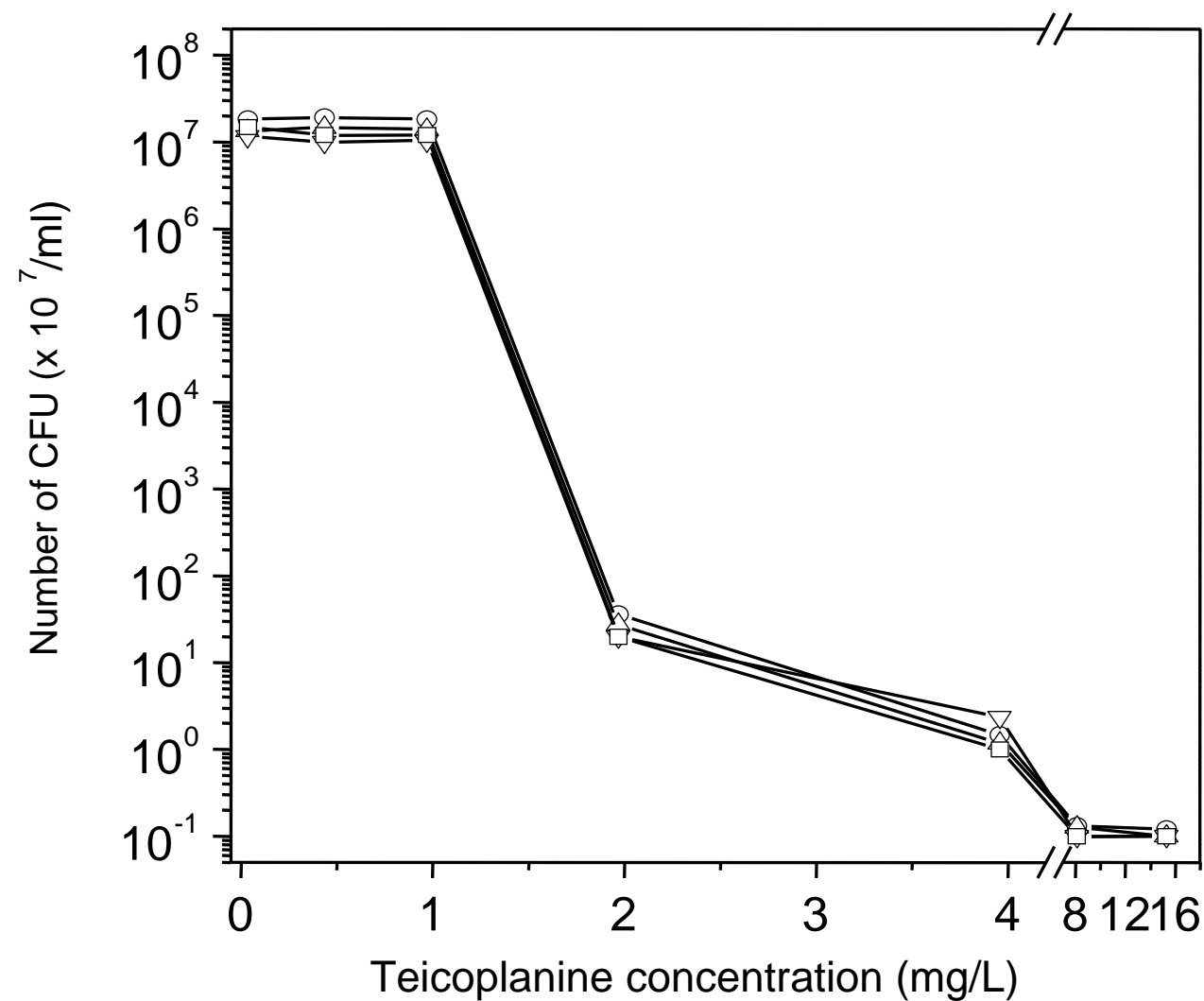

Supplement: Additional file 1 — Figure S1. Profile analysis population with teicoplanin showing the h-GISA population. [file 1745-6150-4-1-S1.pdf]

Figure S2

**a**

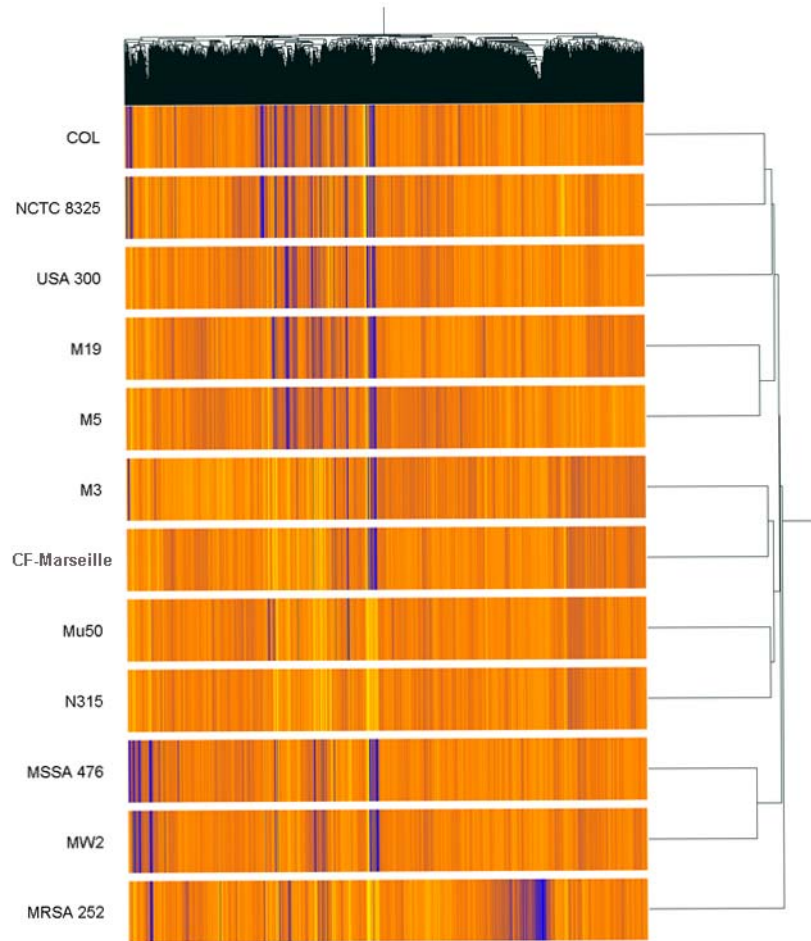

**b**

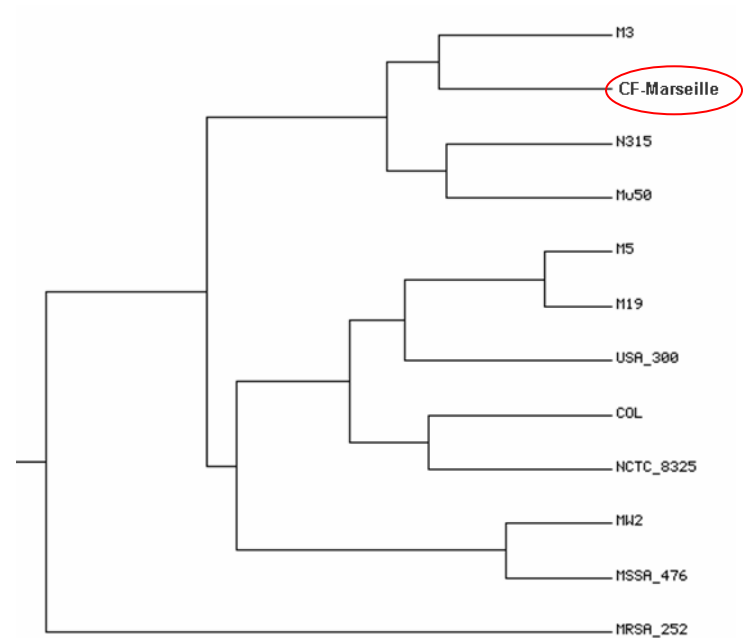

Supplement: Additional file 4 — Figure S2. Complete Genome Hybridization results with other strains. Gene and strain clustering by GeneSpring (a). Dendogram based on the presence and absence of genes in the 12 strains on CGH results (b). [file 1745-6150-4-1-S4.pdf]
