# Supplementary material for: Genomic analysis of an emerging multiresistant Staphylococcus aureus strain rapidly spreading in cystic fibrosis patients revealed the presence of an antibiotic inducible bacteriophage
Source: Biol Direct. 2009 Jan 13;4:1. doi: 10.1186/1745-6150-4-1 (PMC2629466; doi:10.1186/1745-6150-4-1)
Supplement: Additional file 2 — Table S1. List of genes down- and up-regulated in CF-Marseille as compared to other available genomes. [file 1745-6150-4-1-S2.pdf]

Table S1. List of genes down- and up-regulated in CF-Marseille as compared to other available genomes.

**Function**	**Fold change (CF-Marseille/All other)**	**Short name**
**phage DnaC-like protein**	0,0345	SAOUHSC_02216
**CadC**	0,0411	cadC
**CadX**	0,0411	cadX
**Hypothetical protein**	0,114	SAVP033
**Hypothetical protein**	0,125	SAOUHSC_01957
**hypothetical protein (pseudogene)**	0,125	SAS1751
**epidermin immunity protein F**	0,137	epiG
**Hypothetical protein**	0,137	SAOUHSC_01945
**putative lantibiotic ABC transporter protein**	0,137	SAS1739
**Hypothetical protein**	0,235	SAS1909
**phi PVL orf 39-like protein**	0,235	SAV1988
**Hypothetical protein**	0,268	SAOUHSC_02215
**Hypothetical protein**	0,344	SA0956

**putative manganese transport protein**	0,344	SAR1079
**Mn2+/Fe2+ transporter, NRAMP family**	0,344	SACOL1114
**Hypothetical protein**	0,347	SA1793
**CadD**	0,404	cadD
**Hypothetical protein**	0,416	SAV0866
**Hypothetical protein**	0,422	SAR1528
**Hypothetical protein**	2,067	SAS0937
**capsular polysaccharide synthesis enzyme Cap5D**	2,129	cap5D
**potential ATP-binding protein**	2,135	pSAS14
**recombinase Sin**	2,135	sin
**bacteriophage L54a, deoxyuridine 5-triphosphate nucleotidohydrolase**	2,162	SAOUHSC_01552
**prophage L54a, deoxyuridine 5'-triphosphate nucleotidohydrolase**	2,162	dut
**putative dUTP pyrophosphatase**	2,162	SAR2072
**Hypothetical protein**	2,247	SAR1515

**phiSLT ORF104a-like protein, repressor**	2,247	SAUSA300_1434
**putative phage regulatory protein**	2,247	SAS0897
**arsenic efflux pump protein**	2,357	arsB
**phiSLT ORF92-like protein, uncharacterized**		
**phage protein (possible DNA packaging)**	2,4745	SAUSA300_1400
**prophage L54a, DNA packaging protein, putative**	2,4745	SACOL0371
**putative regulatory protein**	2,515	SAR1555
**Hypothetical protein**	2,548	SAS0901
**phiSLT ORF71-like protein**	2,548	SAUSA300_1431
**Hypothetical protein**	2,552	MW0744
**Hypothetical protein**	2,649	SAS0939
**Hypothetical protein**	2,702	MW1397
**capsular polysaccharide synthesis enzyme Cap5E**	2,703	capE
**Hypothetical protein**	2,817	SAR1512
**arsenate reductase**	2,821	arsC
**Immunoglobulin G binding protein A precursor**	3,193	SAOUHSC_00069

**major tail protein**	3,213	SAOUHSC_01529
**phiSLT ORF213-like protein, major tail protein**	3,213	SAUSA300_1397
**prophage L54a, major tail protein, putative**	3,213	SACOL0375
**phiSLT orf 110-like protein**	3,2995	SAOUHSC_01532
**phiSLT ORF110-like protein**	3,2995	SAUSA300_1399
**Hypothetical protein**	3,491	SAR1514
**putative surface anchored protein**	3,508	SAS0520
**Ser-Asp rich fibrinogen-binding bone sialoprotein-binding protein**	3,508	sdrD
**Hypothetical protein**	3,567	SAUSA300_0915
**ABC transporter ATP-binding protein**	3,623	SACOL1352
**Hypothetical protein**	3,623	MW1206
**Hypothetical protein**	3,675	SAS0917
**Hypothetical protein**	4,532	SACOL0372
**Hypothetical protein**	4,855	SAS0896
**pathogenicity island protein**	4,855	SACOL0889

**Replication and maintenance protein**	4,857	SAUSA300_pUSA030006
**Hypothetical protein**	4,976	SAUSA300_0042
**Hypothetical protein**	5,442	SAV2199
**serine protease**	5,621	splA
**phi PVL orf 52-like protein**	5,926	SAOUHSC_01556
**Hypothetical protein**	9,655	MW1412
**Hypothetical protein**	11,01	SAUSA300_pUSA030029
**Hypothetical protein**	13,11	MW0042
**Hypothetical protein**	17,73	SAUSA300_0046
**phiSLT ORF204-like protein**	37,64	SAUSA300_1437
**Hypothetical protein**	49,92	MW0043
**rRNA adenine N-6-methyltransferase (Macrolide-lincosamide-streptogramin B resistance protein)**	129,6	ermC
