# Supplementary material for: Genomic analysis of an emerging multiresistant Staphylococcus aureus strain rapidly spreading in cystic fibrosis patients revealed the presence of an antibiotic inducible bacteriophage
Source: Biol Direct. 2009 Jan 13;4:1. doi: 10.1186/1745-6150-4-1 (PMC2629466; doi:10.1186/1745-6150-4-1)
Supplement: Additional file 3 — Table S2. Patient information and typing of the strains. Study n°: number attributed to the strain in this study. R, resistant; S, sensitive. MecA: detection of the mecA gene. SCC Mec: type of the SCCmec cassette; SCND, cassette type not determined (i.e. not 1, 2 or 4). AGR: agr type. PVL, TSST-1, EXFO-A, EXFO-B: detection of the gene coding for the toxin. [file 1745-6150-4-1-S3.pdf]

Table S2. Patient information and typing of the strains

Study n°: number attributed to the strain in this study. R, resistant; S, sensitive. MecA: detection of the *mec*A gene. SCC Mec: type of the SCC*mec* cassette; SCND, cassette type not determined (i.e. not 1, 2 or 4). AGR: *agr* type. PVL, TSST-1, EXFO-A, EXFO-B: detection of the gene coding for the toxin.

N°	CF	Methicillin	MecA	SCC Mec	AGR	Phage	PVL	TSST -1	EXFO A	EXFO B
M1	No	R	**Pos**	4	1		Neg	Neg	Neg	Neg
M2	No	R	**Pos**	Neg	1	+	Neg	Neg	Neg	Neg
M3	No	R	**Pos**	SCND	2		Neg	Neg	Neg	Neg
M4	No	R	Neg	Neg	1		Neg	Neg	Neg	Neg
M5	No	R	**Pos**	4	1	+	Neg	Neg	Neg	Neg
M6	No	R	**Pos**	4	1		Neg	Neg	Neg	Neg
M7	No	R	**Pos**	4	1	+	Neg	Neg	Neg	Neg
M8	No	R	**Pos**	4	1		Neg	Neg	Neg	Neg
M9	No	R	**Pos**	SCND	2		Neg	Neg	Neg	Neg
M10	No	R	Neg	Neg	2		Neg	Neg	Neg	Neg
M11	Yes	S	Neg	Neg	2		Neg	Neg	Neg	Neg
M12	Yes	S	Neg	Neg	2		Neg	Neg	Neg	Neg
M13	Yes	R	**Pos**	4	1	+	Neg	Neg	Neg	Neg
M14	Yes	R	**Pos**	4	1		Neg	Neg	Neg	Neg
M15	Yes	R	**Pos**	4	1	+	Neg	Neg	Neg	Neg
M16	Yes	R	**Pos**	4	1		Neg	Neg	Neg	Neg
M17	Yes	R	**Pos**	4	1	+	Neg	Neg	Neg	Neg
M18	Yes	R	**Pos**	4	2	+	Neg	Neg	Neg	Neg

M19	Yes	R	**Pos**	4	1	+	Neg	Neg	Neg	Neg
M20	Yes	R	**Pos**	4	1		Neg	Neg	Neg	Neg
M21	Yes	S	Neg	Neg	3		Neg	**Pos**	Neg	Neg
M22	Yes	R	**Pos**	4	1	+	Neg	Neg	Neg	Neg
M23	Yes	S	Neg	Neg	2		Neg	Neg	Neg	Neg
M24	Yes	S	Neg	Neg	3		Neg	Neg	Neg	Neg
M25	Yes	S	Neg	Neg	3		Neg	**Pos**	Neg	Neg
M26	Yes	R	**Pos**	4	1		Neg	Neg	Neg	Neg
M27	Yes	R	**Pos**	1	1		Neg	Neg	Neg	Neg
M28	Yes	R	**Pos**	2	2	+	Neg	Neg	Neg	Neg
M29	Yes	R	**Pos**	4	1	+	Neg	Neg	Neg	Neg
M30	Yes	R	**Pos**	1	2		Neg	**Pos**	Neg	Neg
CF-Marseille	Yes	R	**Pos**	4	2	+	Neg	Neg	Neg	Neg
M32	Yes	R	**Pos**	4	2	+	Neg	Neg	Neg	Neg
M33	Yes	R	**Pos**	4	1	+	Neg	Neg	Neg	Neg
M34	Yes	R	**Pos**	4	1	+	Neg	Neg	Neg	Neg
M35	Yes	S	Neg	Neg	3		Neg	**Pos**	Neg	Neg
M36	Yes	S	Neg	Neg	3		Neg	**Pos**	Neg	Neg
M37	Yes	S	Neg	Neg	2		Neg	Neg	Neg	Neg
M38	Yes	R	**Pos**	4	1	+	Neg	Neg	Neg	Neg
M39	Yes	R	**Pos**	4	1	+	Neg	Neg	Neg	Neg
M40	Yes	R	**Pos**	4	1		Neg	Neg	Neg	Neg
